# Crystal structure of 3,6-dihy­droxy-4,5-di­methyl­benzene-1,2-dicarbaldehyde

**DOI:** 10.1107/S2056989018012495

**Published:** 2018-09-14

**Authors:** Shu Yamazaki, Kazuki Nishiyama, Shiomi Yagi, Tomoyuki Haraguchi, Takashiro Akitsu

**Affiliations:** a Department of Chemistry, Faculty of Science, Tokyo University of Science, 1-3 Kagurazaka, Shinjuku-ku, Tokyo 162-8601, Japan

**Keywords:** crystal structure, alkyl­ating agents, chiral crystallization

## Abstract

The title compound, C_10_H_10_O_4_, was synthesized from tetra­methyl-1,4-benzo­quinone. In the crystal, the almost planar mol­ecule (r.m.s. deviation = 0.024 Å) forms intra­molecular hydrogen bonds between the aldehyde and hy­droxy groups and exhibits *C*_2v_ symmetry. This achiral mol­ecule crystallizes in the chiral space group *P*2_1_ with inter­molecular O—H⋯O and C—H⋯O hydrogen bonding and C—H⋯π and C=O⋯π inter­actions stabilizing the crystal packing.

## Abstract 2

The planar achiral title compound has *C*_2v_ symmetry and crystallizes in the chiral space group *P*2_1_.

## Chemical context   

A number of benzo- and naphtho­quinone derivatives with one or two side chains being capable of alkyl­ation after reduction were found to exhibit inhibitory activity against the growth of transplantable tumours in mice. Furthermore, inhibition of nucleic acid biosynthesis and of the activities of coenzyme Q mediated enzyme systems are also known for related compounds composed of 3,6-dihy­droxy-4,5-di­methyl­benzene-1,2-dicarbaldehyde (Lin & Loo, 1977 ▸; Lin *et al.*, 1978 ▸). According to the literature, these compounds are synthesized from tetra­methyl-1,4-benzo­quinone (Lin & Loo, 1977 ▸; Lin *et al.*, 1978 ▸). Here we report the mol­ecular and crystal structure of an achiral derviative crystallizing in a chiral space group.
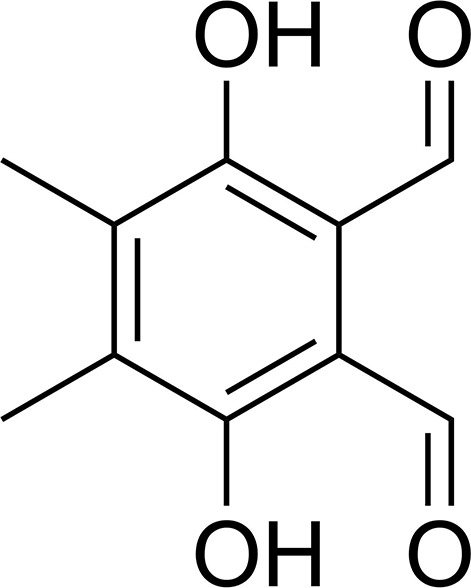


## Structural commentary   

**Figure 1:**
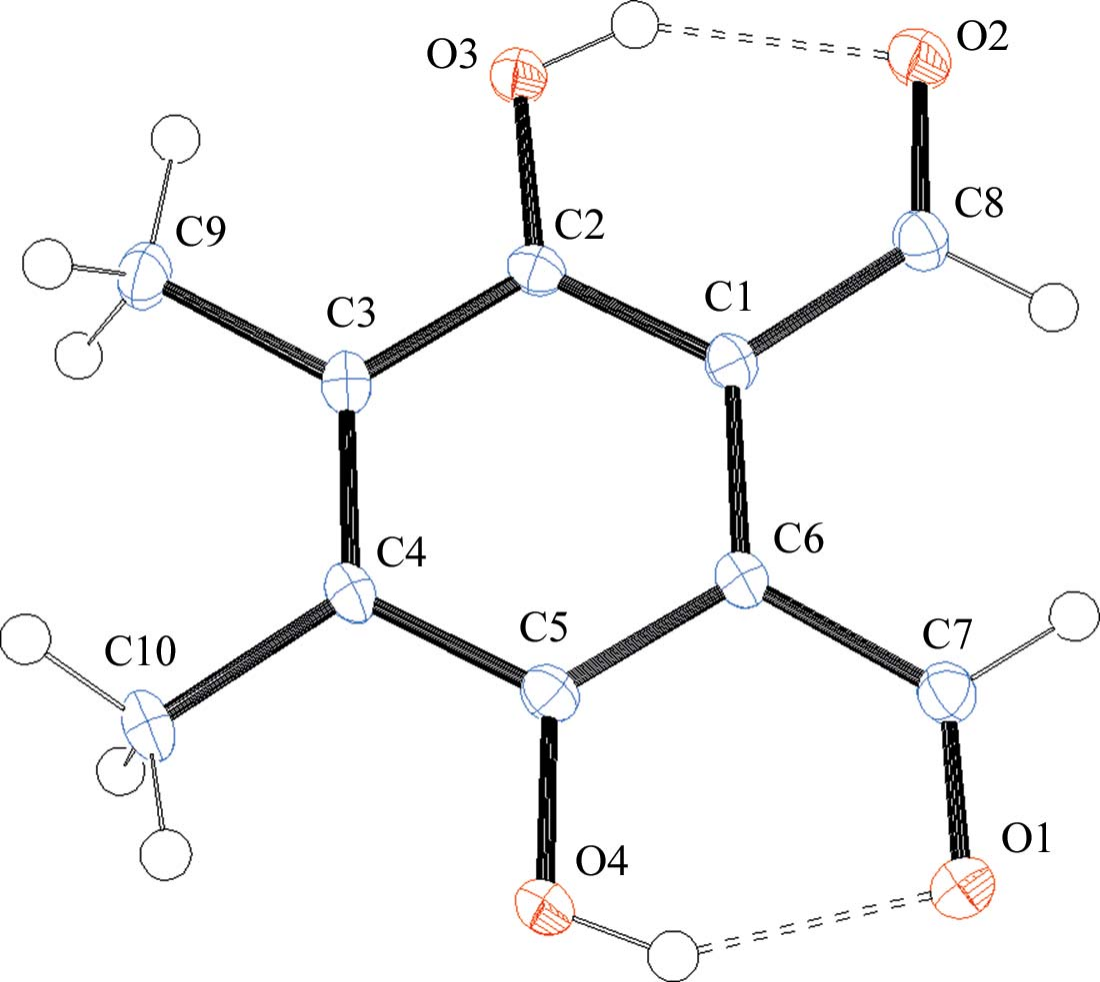
The mol­ecular structure of the title compound, showing the atom-numbering scheme and displacement ellipsoids drawn at the 50% probability level.

The mol­ecular structure of the title compound consists of a benzene ring substituted by two methyl groups, two hy­droxy groups and two aldehyde groups (Fig. 1 ▸). The mol­ecular point group symmetry is *C*_2v_ (H atoms excluded). The C—C bond lengths of the methyl substituents are 1.511 (2) and 1.508 (2) Å, the C—O bond lengths of the hy­droxy substituents are 1.354 (2) and 1.350 (2) Å, and the C—C bond lengths of the aldehyde substituents are 1.464 (2) and 1.462 (2) Å. Two intra­molecular O—H⋯H hydrogen bonds between the hy­droxy and aldehyde functions are observed (Table 1 ▸ and Fig. 1 ▸). The mol­ecule is essentially planar (r.m.s. deviation = 0.024 Å), with the largest deviation found for atom O2 [0.047 (1) Å].

## Supra­molecular features   

**Figure 2:**
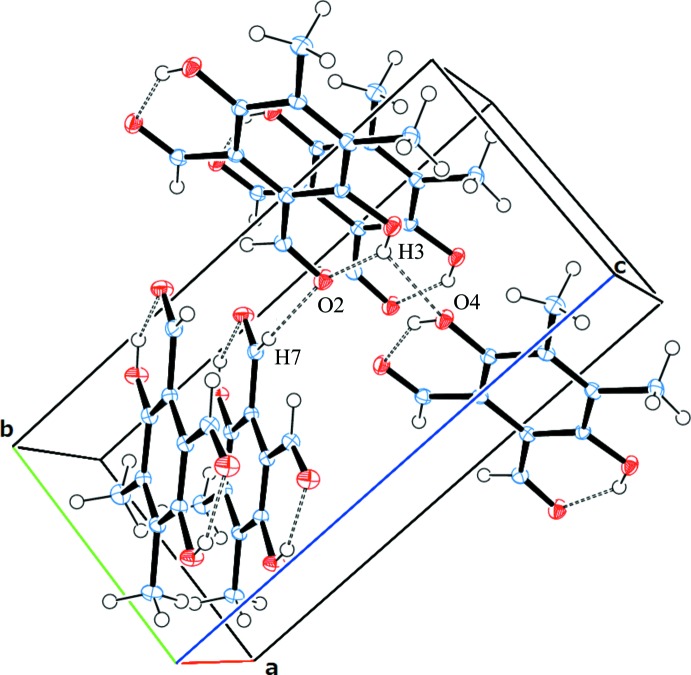
A view of the O—H⋯O hydrogen bonds (dashed lines) present in the crystal lattice of the title compound.

In the crystal, mol­ecules are connected along the *b* axis by O—H⋯O hydrogen bonds and along the *c* axis by C—H⋯O hydrogen bonds (Table 1 ▸ and Fig. 2 ▸). As a result, chiral crystals composed of achiral mol­ecules are formed. Many examples of such chiral crystals forming from achiral mol­ecules have been reported for decades, but the prediction of chiral crystallization is still impossible (Koshima & Matsuura, 1998 ▸; Matsuura & Koshima, 2005 ▸).

The C8=O2 carbonyl group is stacked on top of the aromatic ring, with the O2⋯*Cg*1 distance being 3.4846 (19) Å (*Cg*1 is the centroid of ring C1–C6).

**Figure 3:**
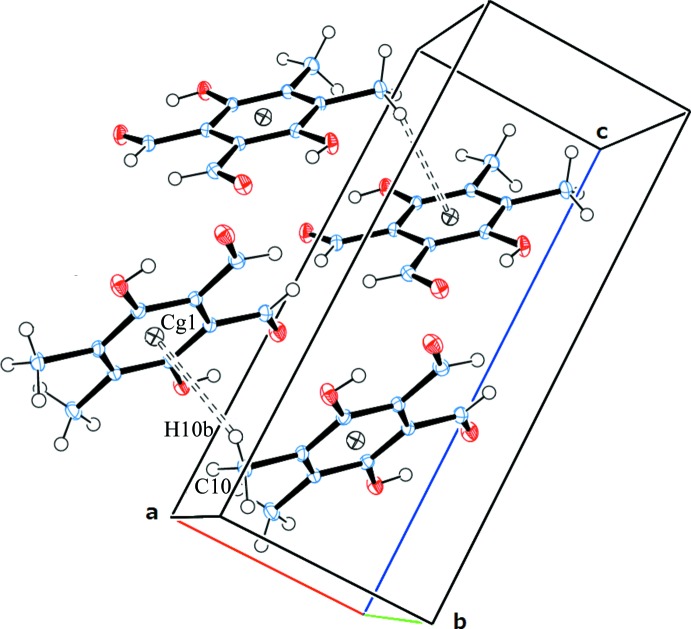
Part of the crystal packing showing the C—H⋯π stacking inter­actions.

In addition, a weak C—H⋯π inter­action C10—H10*B*⋯*Cg*1 (3.131 Å) is also found (Table 1 ▸ and Fig. 3 ▸).

## Database survey   

A search in the Cambridge Structural Database (CSD, Version 5.39, update May 2018; Groom *et al.*, 2016 ▸) for similar 1,2-dicarbaldehyde structures returned five relevant entries: benzene-1,2-dicarbaldehyde [IHEMAJ (Britton, 2002 ▸) and IHEMAJ01 (Mendenhall *et al.*, 2003 ▸)], naphthalene-1,2-dicarbaldehyde (FIYQOT; Britton, 1999 ▸), a chromene-5,6-dicarbaldehyde derivative (IDUCUH; am Ende *et al.*, 2013 ▸) and a cobalt benzene-1,2-dicarbaldehyde complex (JUKZAQ; Lenges *et al.*, 1999 ▸). In the first four structures, the aldehyde functions show C—H⋯O inter­actions (H⋯O distances from 2.226 to 2.360 Å). This is not the case for the cobalt complex JUKZAQ, where the two aldehyde O atoms are facing each other and complexed with cobalt, nor with the title compound where the two aldehyde O atoms are involved in intra­molecular hydrogen bonds and the two aldehyde H atoms are facing each other.

The intra­molecular O—H⋯O inter­action between the 1-carbaldehyde and 2-hy­droxy groups is also observed in compounds such as 1,8-dihy­droxy-2-naphthaldehyde (BABXUA; Peng *et al.*, 2015 ▸) and 2,4,6-tri­hydroxy­benzene-1,3,5-tricarbaldehyde (WEPPUE; von Richthofen *et al.*, 2013 ▸).

## Synthesis and crystallization   

A mixture of tetra­methyl-1,4-benzo­quinone (2.0406 g, 12.4 mmol) and concentrated piperidine (98.0%, 35 ml) was stirred at room temperature for 35 h. The mixture was evaporated and a white inter­mediate product was obtained. To a solution of the obtained inter­mediate product dissolved in acetic acid (18 ml), a mixture of CrO_3_ (1.77 g) and 50% acetic acid (35 ml) was added dropwise at 353 K. After 10 min, the reaction mixture was poured onto crushed ice (100 g). The solution was filtered by vacuum filtration and a crude compound was obtained. The crude compound was dissolved in toluene and purified by silica column chromatography to afford 0.567 g (yield 23.5%) of the title compound as a yellow solid (single crystals served for X-ray analysis). IR (KBr, cm^−1^): 1633 (*s*), 3436 (*m*).

## Refinement   

Crystal data, data collection and structure refinement details are summarized in Table 2 ▸. All H atoms were located in difference Fourier maps. C-bound H atoms were constrained using a riding model [C—H = 0.98 Å and *U*_iso_(H) = 1.5*U*_eq_(C) for methyl H atoms, and C—H = 0.95 Å and *U*_iso_(H) = 1.2*U*_eq_(C) for the aldehyde H atoms]. O-bound H atoms were constrained using a riding model [O—H = 0.84 Å and *U*_iso_(H) = 1.5*U*_eq_(O) for hy­droxy H atoms].

## Supplementary Material

Crystal structure: contains datablock(s) global, I. DOI: 10.1107/S2056989018012495/vm2211sup1.cif

Structure factors: contains datablock(s) I. DOI: 10.1107/S2056989018012495/vm2211Isup2.hkl

Supporting information file. DOI: 10.1107/S2056989018012495/vm2211Isup3.cml

CCDC reference: 1865811

Additional supporting information:  crystallographic information; 3D view; checkCIF report

## Figures and Tables

**Table 1:** Hydrogen-bond geometry (Å, °) *Cg*1 is the centroid of ring C1–C6.

*D*—H⋯*A*	*D*—H	H⋯*A*	*D*⋯*A*	*D*—H⋯*A*
O3—H3⋯O2	0.84	1.87	2.6079 (19)	146
O4—H4⋯O1	0.84	1.82	2.5592 (19)	146
O3—H3⋯O4^i^	0.84	2.40	3.036 (2)	133
O4—H4⋯O2^ii^	0.84	2.34	2.809 (2)	116
C7—H7⋯O2^iii^	0.95	2.51	3.427 (2)	162
C10—H10*B*⋯*Cg*1^iv^	0.98	3.13	3.645	114

Symmetry codes: (i) 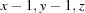; (ii) 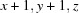; (iii) 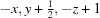; (iv) 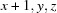.

**Table 2:** Experimental details

Crystal data	
Chemical formula	C_10_H_10_O_4_
*M*_r_	194.18
Crystal system, space group	Monoclinic, *P*2_1_
Temperature (K)	100
*a*, *b*, *c* (Å)	5.251 (2), 6.317 (2), 12.999 (5)
β (°)	91.643 (4)
*V* (Å^3^)	431.0 (3)
*Z*	2
Radiation type	Mo *K*α
μ (mm^−1^)	0.12
Crystal size (mm)	0.38 × 0.30 × 0.13

Data collection	
Diffractometer	Bruker APEXII CCD
Absorption correction	Multi-scan (*SADABS*; Bruker, 2001 ▸)
*T*_min_, *T*_max_	0.785, 0.785
No. of measured, independent and observed [*I* > 2σ(*I*)] reflections	2328, 1228, 1207
*R*_int_	0.014
(sin θ/λ)_max_ (Å^−1^)	0.650

Refinement	
*R*[*F* ^2^ > 2σ(*F* ^2^)], *wR*(*F* ^2^), *S*	0.029, 0.082, 1.06
No. of reflections	1228
No. of parameters	131
No. of restraints	1
H-atom treatment	H-atom parameters constrained
Δρ_max_, Δρ_min_ (e Å^−3^)	0.25, −0.22
Absolute structure	Flack *x* determined using 179 quotients [(*I* ^+^)−(*I* ^−^)]/[(*I* ^+^)+(*I* ^−^)] (Parsons *et al.*, 2013 ▸)
Absolute structure parameter	0.5 (6)

Computer programs: *APEX2* and *SAINT* (Bruker, 2014 ▸), *SHELXT2014* (Sheldrick, 2015*a* ▸), *SHELXL2014* (Sheldrick, 2015*b* ▸), *SHELXTL* (Sheldrick, 2008 ▸), *WinGX* (Farrugia, 2012 ▸), *Mercury* (Macrae *et al.*, 2006 ▸) and *publCIF* (Westrip, 2010 ▸).

## supplementary crystallographic information

### Crystal data

**Table:**

C_10_H_10_O_4_	*F*(000) = 204
*M**_r_* = 194.18	*D*_x_ = 1.496 Mg m^−^^3^
Monoclinic, *P*2_1_	Mo *K*α radiation, λ = 0.71073 Å
*a* = 5.251 (2) Å	Cell parameters from 2083 reflections
*b* = 6.317 (2) Å	θ = 3.1–27.5°
*c* = 12.999 (5) Å	µ = 0.12 mm^−^^1^
β = 91.643 (4)°	*T* = 100 K
*V* = 431.0 (3) Å^3^	Prism, yellow
*Z* = 2	0.38 × 0.30 × 0.13 mm

### Data collection

**Table:**

Bruker APEXII CCD diffractometer	1228 independent reflections
Radiation source: fine-focus sealed tube	1207 reflections with *I* > 2σ(*I*)
Detector resolution: 8.3333 pixels mm^-1^	*R*_int_ = 0.014
φ and ω scans	θ_max_ = 27.5°, θ_min_ = 1.6°
Absorption correction: multi-scan (SADABS; Bruker, 2001)	*h* = −6→6
*T*_min_ = 0.785, *T*_max_ = 0.785	*k* = −3→8
2328 measured reflections	*l* = −16→16

### Refinement

**Table:**

Refinement on *F*^2^	Hydrogen site location: inferred from neighbouring sites
Least-squares matrix: full	H-atom parameters constrained
*R*[*F*^2^ > 2σ(*F*^2^)] = 0.029	*w* = 1/[σ^2^(*F*_o_^2^) + (0.0523*P*)^2^ + 0.0966*P*] where *P* = (*F*_o_^2^ + 2*F*_c_^2^)/3
*wR*(*F*^2^) = 0.082	(Δ/σ)_max_ < 0.001
*S* = 1.06	Δρ_max_ = 0.25 e Å^−^^3^
1228 reflections	Δρ_min_ = −0.22 e Å^−^^3^
131 parameters	Absolute structure: Flack *x* determined using 179 quotients [(I+)-(I-)]/[(I+)+(I-)] (Parsons *et al.*, 2013)
1 restraint	Absolute structure parameter: 0.5 (6)

### Special details

**Table:**

Geometry. All esds (except the esd in the dihedral angle between two l.s. planes) are estimated using the full covariance matrix. The cell esds are taken into account individually in the estimation of esds in distances, angles and torsion angles; correlations between esds in cell parameters are only used when they are defined by crystal symmetry. An approximate (isotropic) treatment of cell esds is used for estimating esds involving l.s. planes.

### Fractional atomic coordinates and isotropic or equivalent isotropic displacement parameters (Å^2^)

**Table:**

	*x*	*y*	*z*	*U*_iso_*/*U*_eq_	
O4	0.7944 (2)	0.8239 (2)	0.27690 (9)	0.0183 (3)	
H4	0.7357	0.8998	0.3237	0.027*	
O2	−0.0040 (2)	0.1772 (2)	0.38214 (9)	0.0193 (3)	
O1	0.5175 (2)	0.9141 (2)	0.42970 (9)	0.0190 (3)	
O3	0.2530 (2)	0.0865 (2)	0.21917 (9)	0.0174 (3)	
H3	0.1418	0.0689	0.2635	0.026*	
C3	0.5775 (3)	0.3220 (3)	0.17026 (11)	0.0136 (3)	
C8	0.1144 (3)	0.3446 (3)	0.38843 (12)	0.0150 (4)	
H8	0.0717	0.4408	0.4414	0.018*	
C4	0.7113 (3)	0.5092 (3)	0.18410 (12)	0.0133 (3)	
C1	0.3173 (3)	0.4049 (3)	0.31924 (12)	0.0128 (3)	
C2	0.3797 (3)	0.2696 (3)	0.23855 (12)	0.0129 (3)	
C7	0.3981 (3)	0.7489 (3)	0.41517 (12)	0.0153 (4)	
H7	0.2619	0.7174	0.4592	0.018*	
C5	0.6521 (3)	0.6468 (3)	0.26629 (12)	0.0134 (4)	
C6	0.4550 (3)	0.5985 (3)	0.33345 (12)	0.0125 (3)	
C9	0.6387 (3)	0.1702 (3)	0.08493 (12)	0.0177 (4)	
H9A	0.6259	0.2441	0.0187	0.027*	
H9B	0.5177	0.0521	0.0848	0.027*	
H9C	0.8122	0.1159	0.0958	0.027*	
C10	0.9192 (3)	0.5739 (3)	0.11251 (13)	0.0179 (4)	
H10A	1.0138	0.4482	0.0915	0.027*	
H10B	1.0356	0.6726	0.1481	0.027*	
H10C	0.8431	0.6429	0.0515	0.027*	

### Atomic displacement parameters (Å^2^)

**Table:**

	*U*^11^	*U*^22^	*U*^33^	*U*^12^	*U*^13^	*U*^23^
O4	0.0187 (6)	0.0167 (7)	0.0198 (6)	−0.0064 (6)	0.0074 (5)	−0.0031 (5)
O2	0.0174 (5)	0.0194 (6)	0.0214 (6)	−0.0060 (6)	0.0049 (4)	−0.0018 (5)
O1	0.0228 (6)	0.0164 (6)	0.0180 (5)	−0.0036 (6)	0.0033 (5)	−0.0035 (5)
O3	0.0179 (5)	0.0158 (6)	0.0186 (6)	−0.0052 (6)	0.0051 (4)	−0.0035 (5)
C3	0.0127 (7)	0.0166 (8)	0.0117 (7)	0.0021 (7)	0.0015 (6)	−0.0001 (7)
C8	0.0130 (7)	0.0172 (9)	0.0148 (7)	−0.0010 (7)	0.0022 (6)	0.0000 (7)
C4	0.0108 (6)	0.0163 (8)	0.0130 (7)	0.0007 (6)	0.0024 (5)	0.0020 (7)
C1	0.0109 (6)	0.0151 (8)	0.0123 (6)	0.0000 (7)	0.0009 (5)	0.0007 (7)
C2	0.0115 (6)	0.0128 (9)	0.0144 (7)	−0.0017 (6)	0.0000 (6)	0.0010 (6)
C7	0.0162 (7)	0.0154 (8)	0.0142 (7)	0.0003 (7)	0.0018 (6)	0.0012 (7)
C5	0.0114 (6)	0.0151 (9)	0.0137 (7)	−0.0014 (7)	0.0001 (5)	0.0011 (7)
C6	0.0115 (6)	0.0139 (8)	0.0120 (7)	0.0001 (6)	0.0011 (5)	0.0011 (7)
C9	0.0185 (7)	0.0192 (9)	0.0157 (7)	0.0000 (7)	0.0034 (6)	−0.0048 (7)
C10	0.0146 (7)	0.0227 (9)	0.0166 (7)	−0.0003 (7)	0.0062 (6)	0.0012 (7)

### Geometric parameters (Å, º)

**Table:**

O4—C5	1.350 (2)	C4—C10	1.511 (2)
O4—H4	0.84	C1—C2	1.399 (2)
O2—C8	1.228 (2)	C1—C6	1.431 (2)
O1—C7	1.229 (2)	C7—C6	1.462 (2)
O3—C2	1.354 (2)	C7—H7	0.95
O3—H3	0.84	C5—C6	1.406 (2)
C3—C4	1.385 (3)	C9—H9A	0.98
C3—C2	1.424 (2)	C9—H9B	0.98
C3—C9	1.508 (2)	C9—H9C	0.98
C8—C1	1.464 (2)	C10—H10A	0.98
C8—H8	0.95	C10—H10B	0.98
C4—C5	1.419 (2)	C10—H10C	0.98
			
C5—O4—H4	109.5	C6—C7—H7	118.4
C2—O3—H3	109.5	O4—C5—C6	122.08 (14)
C4—C3—C2	119.60 (14)	O4—C5—C4	116.85 (13)
C4—C3—C9	121.36 (14)	C6—C5—C4	121.06 (16)
C2—C3—C9	119.04 (16)	C5—C6—C1	118.92 (14)
O2—C8—C1	123.97 (16)	C5—C6—C7	118.66 (16)
O2—C8—H8	118.0	C1—C6—C7	122.42 (13)
C1—C8—H8	118.0	C3—C9—H9A	109.5
C3—C4—C5	119.97 (14)	C3—C9—H9B	109.5
C3—C4—C10	121.60 (15)	H9A—C9—H9B	109.5
C5—C4—C10	118.43 (17)	C3—C9—H9C	109.5
C2—C1—C6	119.38 (13)	H9A—C9—H9C	109.5
C2—C1—C8	119.46 (16)	H9B—C9—H9C	109.5
C6—C1—C8	121.15 (15)	C4—C10—H10A	109.5
O3—C2—C1	122.45 (14)	C4—C10—H10B	109.5
O3—C2—C3	116.47 (14)	H10A—C10—H10B	109.5
C1—C2—C3	121.05 (16)	C4—C10—H10C	109.5
O1—C7—C6	123.30 (15)	H10A—C10—H10C	109.5
O1—C7—H7	118.4	H10B—C10—H10C	109.5
			
C2—C3—C4—C5	0.4 (2)	C3—C4—C5—O4	178.32 (14)
C9—C3—C4—C5	−178.85 (15)	C10—C4—C5—O4	−2.5 (2)
C2—C3—C4—C10	−178.81 (15)	C3—C4—C5—C6	−1.3 (2)
C9—C3—C4—C10	2.0 (2)	C10—C4—C5—C6	177.94 (14)
O2—C8—C1—C2	1.9 (2)	O4—C5—C6—C1	−178.18 (14)
O2—C8—C1—C6	−177.34 (15)	C4—C5—C6—C1	1.4 (2)
C6—C1—C2—O3	−178.32 (13)	O4—C5—C6—C7	2.0 (2)
C8—C1—C2—O3	2.5 (2)	C4—C5—C6—C7	−178.41 (15)
C6—C1—C2—C3	−0.2 (2)	C2—C1—C6—C5	−0.6 (2)
C8—C1—C2—C3	−179.44 (14)	C8—C1—C6—C5	178.56 (15)
C4—C3—C2—O3	178.56 (14)	C2—C1—C6—C7	179.16 (16)
C9—C3—C2—O3	−2.2 (2)	C8—C1—C6—C7	−1.6 (2)
C4—C3—C2—C1	0.4 (2)	O1—C7—C6—C5	−1.6 (2)
C9—C3—C2—C1	179.61 (14)	O1—C7—C6—C1	178.61 (14)

### Hydrogen-bond geometry (Å, º)

Cg1 is the centroid of ring C1-C6.

**Table:**

*D*—H···*A*	*D*—H	H···*A*	*D*···*A*	*D*—H···*A*
O3—H3···O2	0.84	1.87	2.6079 (19)	146
O4—H4···O1	0.84	1.82	2.5592 (19)	146
O3—H3···O4^i^	0.84	2.40	3.036 (2)	133
O4—H4···O2^ii^	0.84	2.34	2.809 (2)	116
C7—H7···O2^iii^	0.95	2.51	3.427 (2)	162
C10—H10···*Cg*1^iv^	0.98	3.13	3.645	114

Symmetry codes: (i) *x*−1, *y*−1, *z*; (ii) *x*+1, *y*+1, *z*; (iii) −*x*, *y*+1/2, −*z*+1; (iv) *x*+1, *y*, *z*.
